# Empiric stereotactic body radiotherapy for presumed early-stage lung cancer: Pulmonary function changes, treatment-related toxicity and survival outcome

**DOI:** 10.1007/s00066-025-02434-8

**Published:** 2025-08-04

**Authors:** Esra Degerli, Karim El-Marouk, Lukas Käsmann, Khulangaa Khaltar, Sina Mansoorian, Cedric Richlitzki, Diego Kauffmann-Guerrero, Amanda Tufman, Niels Reinmuth, Thomas Duell, Nina-Sophie Schmidt-Hegemann, Farkhad Manapov, Claus Belka, Chukwuka Eze

**Affiliations:** 1 Department of Radiation Oncology, LMU University Hospital, LMU Munich, Munich, Germany; 2 German Cancer Consortium (DKTK), partner site Munich; and German Cancer Research Center (DKFZ), Heidelberg, Germany; 3 German Center for Lung Research (DZL), Comprehensive Pneumology Center Munich (CPC-M), Munich, Germany; 4 Department of Medicine V-Pneumology, University Hospital, Ludwig-Maximilians-University Hospital (LMU) Munich, Munich, Germany; 5 Asklepios Fachkliniken Munich, Asklepios Kliniken GmbH, Gauting, Germany; 6 RADIO-LOG Medical Care Centre for Radiation Therapy, 89312 Günzburg, Germany; 7 Bavarian Cancer Research Center (BZKF), Munich, Germany

**Keywords:** Lung function, Lung neoplasms, Radiographic, Stereotactic ablative radiotherapy (SABR), Pathologic confirmation

## Abstract

**Background:**

Due to demographic shifts, the population is aging, and patients are experiencing more comorbidities. Stereotactic body radiotherapy (SBRT) offers high rates of local control for patients with medically inoperable early-stage non-small cell lung cancer (NSCLC). However, obtaining histopathological confirmation can be challenging due to severe comorbidities, small tumors, or unfavorable anatomical locations.

**Methods:**

Between 2011 and 2022, we retrospectively analyzed a cohort of patients who underwent lung SBRT for presumed early-stage NSCLC at our institution. Out of 486 consecutive patients treated during this period, 56 patients (11.5%) with a total of 61 lesions were identified and included in this retrospective study. All included patients lacked histopathological confirmation prior to treatment and had no evidence of other active malignancies. The primary objective of this analysis was to evaluate pulmonary function tests before and after SBRT, including long-term follow-up.

**Results:**

The median overall survival (OS) after empiric SBRT was 50.7 months (95% confidence interval [CI] 12.8–88.7). Survival rates at 1 year and 2 years were 88.4 and 71.1%, respectively. The 1‑, 2‑ and 3‑year local control rates were 96.6%, 92.3% and 87.1%. Pulmonary function tests indicated a relative increase in the mean forced expiratory volume in 1 s (FEV1) of 0.55% (SD 13.5) and 2.0% (SD: 20.0) at 6 and 12 months, respectively. In contrast, the mean diffusing capacity of the lungs for carbon monoxide (DLCO) showed a relative decline of 7.4% (SD 16.6) and 6.3% (SD 26.1) at 6 and 12 months, respectively. Patients with lower comorbidity scores (CCI ≤ 5) exhibited significantly improved OS (*p* = 0.011). Long-term oxygen therapy (LTOT) prior to SBRT was associated with shorter OS (*p* = 0.02) and a relatively high incidence of grade 2–3 pulmonary disorders. Chronic obstructive pulmonary disease (COPD) was identified as a possible risk factor for severe treatment-related toxicity. Notably, all patients who experienced grade 3 pulmonary disorders required LTOT before SBRT.

**Conclusion:**

Empiric SBRT is a safe and effective treatment for presumed early-stage NSCLC in patients without histopathological confirmation. Even in patients requiring oxygen therapy and with severe comorbidities, long-term survival is feasible with acceptable treatment-related toxicity. Optimal dose fractionation and biologically effective dose (BED) levels for frail patients without histological confirmation remain undefined. Prospective trials are warranted to determine the most effective and safe SBRT regimens for this vulnerable patient population.

## Background

Surgical resection is the preferred treatment for stage I and II non-small cell lung cancer (NSCLC). However, when tumor resection is not feasible, local ablative radiotherapy serves as a viable alternative [1, 2]. Several studies have demonstrated that stereotactic radiotherapy (SBRT) is superior to conventional radiotherapy for patients with inoperable stages I–II NSCLC [3–7]. Histological confirmation of tumor tissue is recommended before SBRT. However, many patients with lung tumors are difficult to biopsy due to severe comorbidities, small tumor size, or unfavorable anatomical locations [8, 9]. In the absence of histological confirmation, clinical and imaging features are used to assess the malignancy probability and guide empiric treatment decisions [10, 11]. Several prospective trials, including the ROSEL trial [12], permitted the enrollment of patients without a biopsy. It is essential to assess treatment-related toxicity, especially in vulnerable patients with severe comorbidities such as chronic obstructive pulmonary disease (COPD). While symptomatic lung toxicity from SBRT typically occurs in less than 10% of cases, rates as high as 25% have been reported [13, 14].

Our retrospective study evaluated pulmonary function before and after thoracic SBRT in patients with clinically diagnosed stage I–II lung cancer without histological confirmation. We assessed longitudinal lung function testing, imaging, and clinical follow-up to analyze treatment-related toxicity and clinical outcomes.

## Patients and methods

### Eligibility criteria

For this analysis, we screened all patients aged 18 or older with (presumed) early-stage lung cancer, with or without histopathological confirmation, who received SBRT at our institution between 2011 and 2022. Out of 486 eligible patients, 56 patients treated with empiric SBRT, without pathological confirmation, were included in our retrospective analysis for clinically assumed stage I–II lung cancer, as defined by the Union for International Cancer Control (UICC) 8th edition [15].

The multidisciplinary tumor board, comprising thoracic surgeons, pulmonologists, thoracic oncologists, radiation oncologists, and radiologists, discussed all patients and recommended lung SBRT based on the criteria for malignancy based on the Solitary Pulmonary Nodule (SPN) Malignancy Risk Score [3]. These criteria include age, smoking status, nodule diameter, spiculation, and metabolic activity on 18F-fluorodeoxyglucose positron-emission tomography/computed tomography (18F-FDG-PET/CT) scans [16, 17]. Patients were excluded from our study if they had other active oncological diseases within the past 5 years.

Following approval from our institutional ethics committee (ID: 17-230) and the retrospective nature of this study, informed consent for data collection was retrospectively acquired from all patients who were alive at the time of the analysis.

### Imaging and treatment planning

Prior to SBRT, all patients underwent an 18F-FDG-PET/CT scan to evaluate metabolic activity, and the extent of disease spread. To rule out brain metastases, 15 patients (24.6%) had a cranial magnetic resonance imaging (MRI), while the remaining patients (75.4%) received a cranial CT (cCT) before treatment.

All patients underwent a 4D(four-dimensional)-planning CT. We contoured the gross tumor volume (GTV), and to account for respiratory motion, a margin encompassing all respiratory-phase GTVs was added to the GTV to create the internal target volume (ITV). The planning target volume (PTV) was defined by adding an isotropic margin of 3–6 mm to the ITV. The three irradiation techniques employed for SBRT treatment were three-dimensional conformal radiotherapy (3DCRT), intensity-modulated radiotherapy (IMRT), and volumetric modulated arc therapy (VMAT).

### Follow-up and lung function testing

Patients were advised to undergo routine follow-up examinations at 6 weeks, 3 months, 6 months, and 12 months after SBRT. Following the initial 12 months, evaluations continued at the same intervals. These examinations included regular laboratory evaluations, assessment of Eastern Cooperative Oncology Group (ECOG) performance status, and pulmonary function monitoring through pulmonary function tests (PFTs). PFTs were calculated according the Global Lung Function Initiative (GLI) of the European Respiratory Society (ERS) and included measured forced expiratory volume in 1 s (FEV1), vital capacity (VC), total lung capacity (TLC), diffusing capacity of the lungs for carbon monoxide (DLCO), and carbon monoxide transfer coefficient (K_CO_). The follow-up CT scans were independently evaluated by two board-certified radiation oncologists (LK and SM).

Treatment-related toxicity, including respiratory disorders such as radiation pneumonitis (RP), was evaluated using the Common Terminology Criteria for Adverse Events (CTCAE), version 4 based on each patient’s clinical status and CT scans over 6 months following SBRT. Lung function and respiratory disorders were assessed by comparing baseline and follow-up pulmonary function tests and imaging. Patients with grade 1 respiratory disorders were categorized as asymptomatic (showing findings on imaging only), while grade 2 or higher were classified as symptomatic.

### Statistical analysis

Statistical analysis was performed using SPSS (Version 28, IBM, Armonk, NY, USA) and R (version 4.2.1) using Rstudio (Boston, MA, USA). The median follow-up was calculated using the reverse Kaplan–Meier method, which measures the duration from the completion of SBRT to the most recent follow-up. Overall survival was defined as the time from registration to death from any cause. Local control was defined as the absence of tumor recurrence or progression at the primary treatment site following initial therapy. Survival curves for overall survival, and local control were estimated using the Kaplan–Meier method.

Changes in PFTs after SBRT were compared to the baseline for each patient, and group comparisons were performed using log-rank tests. Most patients had abnormal PFTs, so a proportional change from the baseline was established and evaluated at different intervals using the Wilcoxon signed-rank test. A *p*-value of less than 0.05 was considered statistically significant.

## Results

### Patient and tumor characteristics

Detailed patient and tumor characteristics are provided in Table 1.

**Table 1:** Patient and tumor characteristics

		Median (range):	*N* (%)	Gender	
				Female	Male
**Patient characteristics**					
*Total:*	*–*	*–*	56 (100%)	23 (41.1%)	33 (58.9%)
*Age:*	–	69 years (57–88)	–	69 (58–84)	70 (57–88)
*Tobacco consumption:*	*Total:* *Current* *Former* *Nonsmoker*	–	52 (92.9%) 15 (26.8%) 37 (66.1%) 4 (7.1%)	– 4 (26.7%) 16 (43.2%) 3 (75%)	– 11 (73.3%) 21 (56.8%) 1 (25%)
*Tobacco consumption among smokers (pack–years):*	–	50 (5–120)	–	60 (20–80)	50 (5–120)
*COPD* *GOLD I–II* *GOLD III–IV*	*–*	–	48 (85.7%) 10 (17.9%) 38 (67.9%)	19 (39.6%) 3 (30%) 16 (42.1%)	29 (60.4%) 7 (70%) 22 (57.9%)
*Charlson comorbidity index*	– *0–2* *3–4* *5–9* *≥ 10*	5 (2–13)	– 2 (3.6%) 20 (35.7%) 25 (44.6%) 9 (16.1%)	– 2 (100%) 9 (45%) 11 (44%) 1 (11.1%)	– 0 (0%) 11 (55%) 14 (56%) 8 (88.9%)
**Tumor characteristics (*n* = 61)**					
*Location*	*Peripheral* *Central* *Ultracentral*	–	37 (60.7%) 19 (31.1%) 5 (8.2%)	16 (43.2%) 7 (36.8%) 1 (20%)	21 (56.8%) 12 (63.2%) 4 (80%)
*T category for presumed lung cancer*	*T1* *T2* *T3*	–	49 (80.3%) 7 (11.5%) 5 (8.3%)	21 (42.9%) 3 (42.9%) 0 (0%)	28 (57.1%) 4 (57.1%) 5 (100%)
*UICC stage for presumed lung cancer*	*I* *II*	–	53 (86.9%) 8 (13.1%)	24 (45.3%) 0 (0%)	29 (54.7%) 8 (100%)
*Treatment volumes*	*GTV* *ITV* *PTV*	6.2 cm^3^ (0.5–125.3) 11.2 cm^3^ (1.5–140.3) 35.1 cm^3^ (5.9–251.2)	–	4.9 cm^3^ (0.5–36.6**)** 10.2 cm^3^ (1.5–44.3) 28.2 cm^3^ (5.9–99.8)	7.7 cm^3^ (0.9–125.3) 11.9 cm^3^ (2.1–140.3) 42.6 cm^3^ (9.9–251.2)

*COPD* chronic obstructive pulmonary disease, *GOLD* Global Initiative for Chronic Obstructive Lung Disease, *GTV* gross tumor volume, *ITV* internal target volume, *PTV* planning target volume, *UICC* Union for International Cancer Control

The cohort included 56 patients, 33 of whom (58.9%) were male. The median age at diagnosis for the entire cohort was 69 years (range: 57–88). Tobacco consumption was reported by 52 patients (92.9%), including 15 (26.8%) current smokers and 37 (66.1%) former smokers. The median pack–years among smokers was 50 (range: 5–120). Most patients (48, 85.7%) had chronic obstructive pulmonary disease (COPD), which was classified using the Global Initiative for Chronic Obstructive Lung Disease (GOLD) criteria. Ten patients (17.9%) were classified as GOLD I–II, while 38 (67.9%) were categorized as GOLD III–IV. Prior to SBRT, 23 patients (41.1%) required long-term oxygen therapy, whereas 33 patients (58.9%) did not.

The primary reason for the lack of confirmation was the frailty of our patient population, with a median Charlson Comorbidity Index (CCI) of 5 (range: 2–13). Comorbidity burden varied among patients, with 2 (3.6%) having a score of 0–2 points, 20 (35.7%) having 3–4 points, 25 (44.6%) scoring 5–9 points, and 9 (16.1%) having 10 or more points. Biopsy was attempted in 28 (50%) patients for 32 (52.5%) tumors and all these patients were deemed inoperable. Most tumors were located peripherally (37 tumors, 60.7%), followed by central tumors, which per definition also include ultracentral tumors (24 tumors, 39.3%). According to the UICC classification, the presumptive tumor stage was stage I for 53 tumors (86.9%), with 8 (13.1%) of the tumors being classified as stage II (13.1%).

### Treatment characteristics

Treatment volumes are displayed in Table 1. The biologically effective dose (BED) is determined using the formula: BED = *n* × *d* × [1 + *d*/(α/β)], where “*n*” represents the number of fractions, “*d*” is the dose per fraction, and “α/β” denotes the tissue’s sensitivity to radiation. For tumor, the α/β value is typically considered to be 10 Gy. BED ranged from 67.5 to 120 Gy (median: 95.2 Gy) and was delivered in 1–10 fractions. SBRT protocols included three fractions of 13.5 Gy prescribed to the 65% isodose line, accounting for 44.3% of treatments, primarily for peripheral tumor locations. For central tumor locations, eight fractions of 7.5 Gy prescribed to the 80% isodose line were used in 32.8% of treatments. For ultracentral tumor locations, ten fractions of 5 Gy prescribed to the 80% isodose line were employed, representing 10% of treatments.

### Follow-up, PFT, and lung toxicity

The mean PFT values at baseline and follow-up intervals are presented in Table 2.

**Table 2:** Results of lung function testing before SBRT and during follow-up

	Parameter	Mean value (range)
*Baseline PFTs ≤* *3 months before SBRT* *n (%): 47 (77%)*	FEV1 (liters) FEV1 (% of predicted) FVC (liters) FVC (% of predicted) TLC (liters) TLC (% of predicted) DLCO (mmol/min/kPa) DLCO (% of predicted) K_CO_ (mmol/min/kPa) K_CO_ (% of predicted)	1.4 (0.5–2.3) 55.8 (17.0–101.0) 2.6 (0.7–4.2) 69.8 (26.0–117.0) 7.0 (3.2–15.4) 109.6 (66.6–192.0) 3.8 (1.4–12.7) 47.7 (15.0–219.0) 0.8 (0.3–1.5) 63.0 (25.0–137.0)
*PFTs at 6 weeks* *n (%):34 (55.7%)*	FEV1 (liters) FEV1 (% of predicted) FVC (liters) FVC (% of predicted) TLC (liters) TLC (% of predicted) DLCO (mmol/min/kPa) DLCO (% of predicted) K_CO_ (mmol/min/kPa) K_CO_ (% of predicted)	1.5 (0.5–3.7) 57.3 (16.0–119.4) 2.7 (1.3–5.5) 75.1 (31.0–125.7) 6.7 (2.8–10.1) 110.6 (55.9–145.0) 3.8 (1.5–10.4) 41.2 (18.0–60.1) 0.8 (0.4–1.4) 60.5 (28.5–109.0)
*PFTs at 3 months* *n (%):27 (44.3%)*	FEV1 (liters) FEV1 (% of predicted) FVC (liters) FVC (% of predicted) TLC (liters) TLC (% of predicted) DLCO (mmol/min/kPa) DLCO (% of predicted) K_CO_ (mmol/min/kPa) K_CO_ (% of predicted)	1.5 (0.4–2.6) 60.9 (15.2–111.0) 2.5 (1.1–3.5) 77.2 (29.4–132.0) 6.6 (3.5–10.0) 107.4 (63.6–146.0) 3.5 (1.5–6.2) 45.0 (20.5–79.0) 0.8 (0.4–1.7) 67.7 (28.5–139.6)
*PFTs at 6 months* *n (%):36 (59%)*	FEV1 (liters) FEV1 (% of predicted) FVC (liters) FVC (% of predicted) TLC (liters) TLC (% of predicted) DLCO (mmol/min/kPa) DLCO (% of predicted) K_CO_ (mmol/min/kPa) K_CO_ (% of predicted)	1.6 (0.5–3.9) 57.7 (16.0–111.6) 2.8 (1.3–5.4) 75.5 (31.0–122.6) 6.8 (3.3–10.3) 105.3 (68.9–150.0) 3.6 (1.1–7.6) 43.6 (13.0–112.0) 0.8 (0.3–1.5) 59.6 (20.0–118.8)
*PFTs at 12 months* *n (%):26 (42.6%)*	FEV1 (liters) FEV1 (% of predicted) FVC (liters) FVC (% of predicted) TLC (liters) TLC (% of predicted) DLCO (mmol/min/kPa) DLCO (% of predicted) K_CO_ (mmol/min/kPa) K_CO_ (% of predicted)	1.6 (0.6–3.8) 59.3 (24.0–104.0) 2.7 (1.4–5.1) 80.3 (48.0–137.0) 7.0 (3.3–11.2) 101.0 (63.0–160.0) 3.6 (1.5–10.3) 37.9 (15.0–71.0) 0.8 (0.3–1.4) 64.6 (24.0–132.0)

*DLCO* diffusing capacity of the lungs for carbon monoxide, *FEV1* forced expiratory volume in 1 s, *FVC* forced vital capacity, *K*_*CO*_ carbon monoxide transfer coefficient, *PFT* pulmonary function test, *SBRT* stereotactic body radiotherapy, *TLC* total lung capacity

PFTs were conducted in 34 (55.7%) instances at 6 weeks, 27 (44.3%) instances at 3 months, 36 (59%) instances at 6 months, and 26 (42.6%) instances at 12 months. FEV1 and DLCO were the primary parameters monitored over time following SBRT.

The mean FEV1 for all instances was 1.4 L (range: 0.5–2.3) at baseline (Table 2), 1.5 L (range: 0.5–3.7) at 6 weeks, 1.5 L (range: 0.4–2.7) at 3 months, 1.6 L (range: 0.5–3.9) at 6 months, and 1.6 L (range: 0.6–3.8) at 12 months. The mean DLCO at baseline was 3.8 mmol/min/kPa (range: 1.4–12.7), 3.8 mmol/min/kPa (range: 1.5–10.4) at 6 weeks, 3.5 mmol/min/kPa (range: 1.5–6.2) at 3 months, 3.6 mmol/min/kPa (range: 1.1–7.6) at 6 months, and 3.6 mmol/min/kPa (range: 1.5–10.3) at 12 months. With the exception of a mild decrease in mean FVC (L) after 12 months (*p* = 0.01), none of the changes in PFTs were statistically significant (*p* > 0.05; Table 3).

**Table 3:** Pulmonary function test (PFT) distribution at baseline and 12 months post-stereotactic body radiotherapy (SBRT)

	Baseline (*n* = 47)		12 Mo post-SBRT (*n* = 26)		Relative ∆ from baseline in % (SD)	Wilcoxon signed-rank test: no. of pairs (*p*-value)
	No.	Mean (SD)	No.	Mean (SD)		
*FEV1 (liters)*	46	1.43 (0.6)	23	1.6 (0.7)	2.0 (20.0)	19 (0.75)
*FEV1 (%Predicted)*	46	55.8 (22.7)	23	59.3 (19.1)	6.0 (26.0)	19 (0.63)
*FVC (liters)*	45	2.6 (0.9)	23	2.7 (0.8)	−7.81 (11.7)	**18** **(0.01)**
*FVC (%Predicted)*	45	69.8 (21.9)	23	80.3 (27.3)	8.95 (31.8)	18 (0.71)
*DLCO (mmol/min/kPa)*	35	3.77 (2.0)	17	3.61 (2.0)	−6.33 (26.1)	10 (0.11)
*DLCO (%Predicted)*	35	47.7 (35.9)	17	37.9 (12.7)	−3.37 (29.7)	10 (0.39)

*Bold lettering:* Significant p-value

**Fig. 1:**
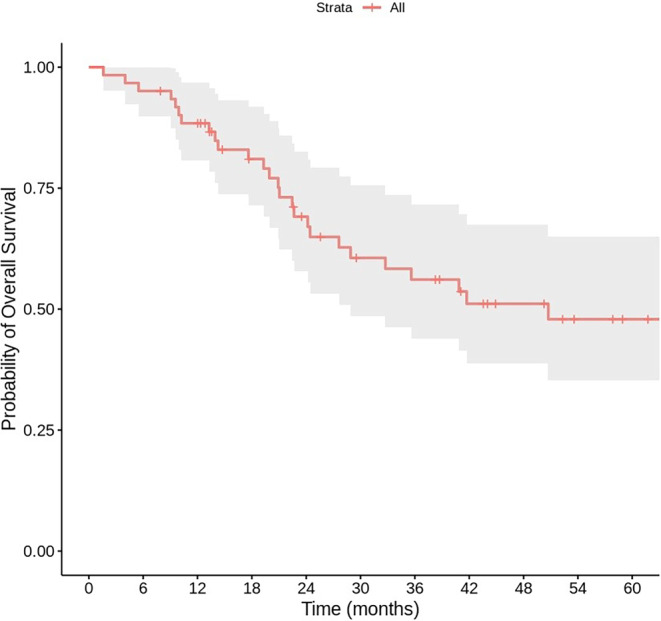
Kaplan–Meier curve for overall survival

**Fig. 2:**
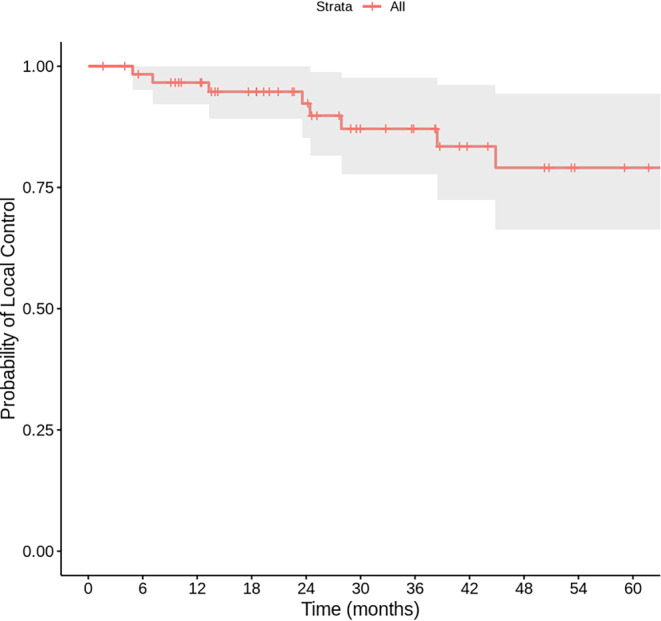
Kaplan–Meier curve for local control

Initially, of the 56 patients eligible for follow-up, 2 died from causes unrelated to the tumor or treatment, and 7 declined follow-up before the 6‑month mark. With a median follow-up of 52.3 months (range: 1.6–123.4 months), the median overall survival was 50.7 months (95% CI: 12.8–88.7). The 1‑year and 2‑year survival rates were 88.4 and 71.1%, respectively (Fig. 1). Overall survival was significantly longer in patients with CCI ≤ 5 and in those without prior long-term oxygen therapy (*p* = 0.011 and *p* = 0.02, respectively). The 1‑, 2‑ and 3‑year local control rates were 96.6%, 92.3% and 87.1%, respectively (Fig. 2). In all, 21 patients (37.5%) experienced respiratory disorders, including RP of grade 2 or higher, with 19 of these classified as grade 3. A total of 19 cases of grade 3 pulmonary disorders were reported, including events such as pneumonitis, pneumonia, and COPD exacerbations. Due to overlapping clinical and imaging findings, a clear distinction between these entities may not have been consistently made. All 19 patients who developed grade 3 respiratory disorders were receiving long-term oxygen therapy before SBRT. In contrast, patients without long-term oxygen therapy experienced only grade 1 or 2 respiratory disorders, with grade 2 events occurring in 3 patients.

## Discussion

Lung cancer is the most commonly diagnosed cancer worldwide and the leading cause of cancer-related deaths representing a serious medical condition, especially among men, with nearly 2.5 million cases and 1.8 million deaths each year [18, 19]. For early-stage NSCLC, surgery is the preferred treatment option [20, 21]. However, when surgery is not feasible, SBRT is an established alternative, leading to a median overall survival of more than 65 months and high rates of local control in medically inoperable NSCLC, according to recent studies [22, 23]. Histopathological confirmation of lung cancer is recommended before treatment. However, many patients cannot undergo a biopsy due to severe health issues, small tumor size, or challenging tumor locations [24]. In such cases, clinical and imaging features including positron-emission tomography (PET)/computed tomography (CT) imaging and risk scores guide treatment without requiring biopsy confirmation [25]. Notably, the use of empiric lung SBRT has significantly increased over the last decade [24, 26, 27]. Even within the same healthcare system, the use of lung SBRT without a preceding biopsy varies widely, ranging from 0 to 61% across medical centers [28]. The consensus-based recommendation in the S3 guidelines of the German Guideline Program in Oncology recommends offering stereotactic ablative radiotherapy directly if an interdisciplinary consensus determines that the risk of biopsy is too high [29]. Several studies have explored the outcomes of empiric SBRT for presumed early-stage lung cancer. However, treatment-related side effects and detailed evaluations of changes in lung function within this highly vulnerable patient population remain inadequately addressed. Only a few comprehensive studies have examined the results of empiric SBRT for lung tumors [27, 28]. According to the US Veterans Affairs health care system, 2221 patients treated with SBRT for cT1–T2a cN0 cM0 NSCLC were evaluated, of which 330 (14.9%) were treated empirically, similar to the utilization rate of our study [28]. Interestingly, no significant differences were found between the histopathologically confirmed and the assumed NSCLC groups regarding age, gender, tobacco consumption, and comorbidities. Their study found that patients in the pathological confirmation group were more likely to have T2 tumors (*p* < 0.05) and reside within the histoplasmosis belt compared to the nonpathological confirmation group (*p* < 0.05), while overall survival was negatively associated with T2a tumor stage, and CCI, and lung cancer-specific survival was adversely affected by T2a tumor stage, lack of PET scan use, and being in the pathological confirmation group.

Furthermore, overall survival was similar in both groups, with 34 months compared to 37 months (*p* = 0.29). Notably, fractionation concepts were not reported and may vary based on tumor location, size, medical center, and treatment date. Lung function testing was also not reported [28]. Another cohort from the Netherlands assessed 591 patients, revealing a significantly higher rate of empiric SBRT utilization at 65% [27]. SBRT concepts were reported, delivering 60 Gy in 3–8 fractions (BED_10_ ranging from 105–180 Gy). Furthermore, the outcomes for histopathologically confirmed versus presumed NSCLC were similar, with a median overall survival of 39.2 months compared to 40.2 months, respectively. Severe treatment-related toxicity (grade 3+) was rarely observed in the entire cohort. A total of 18 patients (3%) developed grade 3 + RP in the combined cohort; however, no significant difference was found or reported between the two cohorts. Patients with a clinical diagnosis had significantly smaller tumors (*p* < 0.001) and worse pulmonary function (*p* = 0.025) than those with a pathological diagnosis. However, overall survival, regional and distant control, and treatment-related side effects showed no significant differences between the groups [27]. A Japanese study of 173 patients with an empiric SBRT utilization rate of 33% found a similar 3‑year overall survival rate of 54% compared to 57% (*p* = 0.48) but reported no treatment-related grade 4 or 5 radiation pneumonitis [30]. A Chinese multicenter study evaluated 90 patients after propensity score matching between biopsy-proven and clinically diagnosed early-stage NSCLC, of which 45 (50%) patients were treated empirically [31]. Regarding outcomes, the 3‑year local control and overall survival were 92.6%/93% and 84.4%/88.8%, the 5‑year local control and overall survival (OS) rates 85.5%/89.8%, and 63.2%/76.1%, respectively. Relevant treatment-related toxicity (grade 2+ respiratory disorders) occurred in 4 patients (9%) in the cohort without histopathological confirmation. Lung function was not included in routine follow-up for the study. In a small investigation by Haider et al., 23 patients with clinically diagnosed early NSCLC were examined [32]. The median OS was 30.2 months, and severe toxicity (grade 3+) was not reported. In fact, 2 patients (8.7%) experienced grade 1–2 acute side effects, while 3 patients (13%) had grade 1–2 late side effects. However, results and rates of lung function were not reported [32].

The CCI and the necessity for LTOT emerged as significant predictors of overall survival (OS) in our study cohort. With a median CCI of 5 (range: 2–13), our findings underscore the considerable comorbidity burden among these patients, emphasizing the challenges of treating this vulnerable population. Patients with lower comorbidity scores (CCI ≤ 5) exhibited significantly improved OS (*p* = 0.011) compared to those with CCI > 5, reaffirming the prognostic importance of comorbidities in this context. Moreover, LTOT prior to SBRT was associated with shorter OS (*p* = 0.02) and a relatively high incidence of grade 2–3 respiratory disorders. Notably, only 3 (14%) of the patients who developed grade 2+ respiratory disorders did not require LTOT before SBRT, reflecting the adverse effect of severely diminished pulmonary function on treatment-related toxicity. Interestingly, other studies examining empiric SBRT have not documented the prevalence of LTOT in their patient cohorts [28, 30, 31]. Our findings underscore the importance of comprehensive pretreatment assessments, which include thorough evaluations of comorbidities and respiratory support requirements, to enhance patient stratification and optimize management strategies. COPD was identified as a possible risk factor for severe treatment-related toxicity, including radiation pneumonitis, with nearly all patients who developed grade 2+ respiratory disorders having a diagnosis of COPD (21 patients, 95.46%). Furthermore, patients requiring LTOT prior to SBRT were at a significant increased risk of severe treatment-related toxicity. Notably, all patients who experienced grade 3+ respiratory disorders required LTOT. Therefore, involving these patients in a shared decision-making process is crucial, as it ensures they are well-informed about the potential risks associated with treatment and the importance of subsequent follow-up care.

Our study observed a significantly higher incidence of respiratory disorders such as RP within 6 months after SBRT compared to the rates reported in the literature reviewed by Berman et al. Specifically, 21 patients in our cohort experienced grade 2+ respiratory disorder, with a concerning 19 cases classified as grade 3. Importantly, no grade 4 or 5 respiratory disorders events occurred. In contrast, the studies cited by Berman et al. demonstrated lower RP rates: Sakanaka et al. observed 5.4% grade 2 RP, Yoshitake et al. reported 2.3% grade 2 RP, and Inoue et al. reported 8.8% grade 2, 5.3% grade 3, and 1.8% grade 5 RP, Wang et al. also reported lower rates with 8% grade 2 and 8% grade 3 RP [9]. A crucial limitation in comparing our findings directly is the lack of detailed patient characteristics, such as LTOT or COPD, within the studies reviewed by Berman et al. While our analysis suggests LTOT and COPD as potential contributing factors to the observed discrepancy, further investigation is warranted to elucidate the specific factors influencing RP development following SBRT.

In addition to LTOT and COPD, Dover et al. emphasized tumor location as a potential risk factor for other toxicities, particularly when the tumor is centrally located. Central or ultracentral tumors can increase the risk of esophageal toxicity [33]. In our cohort, 4 patients experienced dysphagia. Three patients had grade 1 dysphagia, and 1 patient had grade 2 dysphagia. Notably, the patient with grade 2 dysphagia had a peripheral tumor, while 2 of the grade 1 dysphagia cases were associated with central tumors and 1 with an ultracentral tumor. Several studies have investigated the use of SBRT with a BED_10_ < 90 Gy in order to reduce the risk of treatment-related toxicity, particularly in elderly or frail patients with significant comorbidities and limited life expectancy [34, 35]. These studies demonstrated that acceptable local control can still be achieved with reduced dose regimens in selected patient populations.

Nevertheless, it is important to note that a BED_10_ ≥ 100 Gy remains the established standard of care for medically fit patients, as it is associated with improved tumor control outcomes [20]. However, in clinical reality, individualized treatment approaches are often necessary to balance efficacy and safety.

In our study, where many patients had compromised general health and were treated with heterogeneous dose schedules—including some with BED_10_ < 90 Gy—we observed local recurrences in 10 lesions, resulting in a local control rate of 96.6%, 92.3%, 87.1% after 1‑, 2‑, and 3‑years, respectively. While these rates are slightly lower than those reported in studies using higher BEDs, they still demonstrate meaningful local disease control, particularly considering the frailty of the treated population. Our findings support the notion that in selected high-risk patients, SBRT with reduced BED may offer a reasonable compromise between efficacy and tolerability.

Importantly, if clinically justified, a biopsy attempt should always be performed. However, not all patients will undergo the procedure due to life-threatening risks. A multidisciplinary tumor board recommendation for empiric SBRT should be made collaboratively by thoracic surgeons, radiologists, pulmonologists, and radiation oncologists. Additionally, after SBRT, all patients should be encouraged to pursue routine clinical follow-up, including lung function testing. According to current literature, the decrease in DLCO (%) is the most sensitive parameter for the early detection of RP.

Therefore, all patients should undergo routine testing to identify those at risk and start treatment early to prevent severe or life-threatening complications. While providing valuable insights into the treatment-related toxicity and outcomes of empiric SBRT in presumed early-stage NSCLC patients, our study has several limitations. The rate of empiric therapy matches what is reported in the literature, but the relatively small sample size and potential selection bias must be taken into account. Additionally, the retrospective design and the limited number of complete pulmonary function tests due to the frailty and comorbidities of the study population restrict the statistical power of our analysis and limit the generalizability of our findings to the broader population of patients undergoing empiric SBRT. Despite these significant limitations, we believe that our study contributes to the clinical evidence of empiric SBRT in patients without histopathological confirmation of early-stage NSCLC, identifies high-risk patients, and supports patient stratification and personalized treatment. The focus of the current study is clinical outcome and longitudinal pulmonary function analysis/changes. A detailed follow-up analysis with extensive dissection of patterns of relapse analysis is in preparation.

## Conclusion

Stereotactic body radiotherapy (SBRT) for patients without histopathological confirmation of early-stage non-small cell lung cancer is a safe and effective treatment that can lead to long-term survival. A thorough diagnostic and treatment workup and tumor board discussion is essential when performing lung SBRT for this highly vulnerable patient group. Even in individuals requiring long-term oxygen therapy, empiric SBRT remains a viable option, with an acceptable rate of treatment-related toxicity after careful consideration within shared decision-making. Optimal dose fractionation and biologically effective dose levels for frail patients without histological confirmation remain undefined. Prospective trials are warranted to determine the most effective and safe SBRT regimens for this vulnerable patient population.
